# Vacuum Based Gas Sensing Material Characterization System for Precise and Simultaneous Measurement of Optical and Electrical Responses

**DOI:** 10.3390/s22031014

**Published:** 2022-01-28

**Authors:** Jie Wei, Meng Zhao, Cong Wang, Jun Wang, Jian-Min Ye, Yu-Chen Wei, Zhe-Yi Li, Run Zhao, Guo-Zhen Liu, Yan-Hong Geng, Rui Wang, Hui-Dong Xiao, Ying Li, Chao-Ya Li, Zhi-Qiang Gao, Ju Gao

**Affiliations:** 1 Jiangsu Key Laboratory of Micro and Nano Heat Fluid Flow Technology and Energy Application, Suzhou University of Science and Technology, Suzhou 215009, China; Weijie0621@post.usts.edu.cn (J.W.); yejianmin@post.usts.edu.cn (J.-M.Y.); liying@post.usts.edu.cn (Y.L.); lichaoya@post.usts.edu.cn (C.-Y.L.); 2 School of Information and Communication, Harbin Institute of Technology, Harbin 150001, China; weiyuchen@hit.edu.cn (Y.-C.W.); 21S005003@stu.hit.edu.cn (Z.-Y.L.); gao_zhiqiang@hit.edu.cn (Z.-Q.G.); 3 School of Physical Science and Technology, Suzhou University of Science and Technology, Suzhou 215009, China; wjk31@163.com (J.W.); zr@usts.edu.cn (R.Z.); guozhen.liu@hotmail.com (G.-Z.L.); jugao@hku.hk (J.G.); 4 Suzhou Institute of Metrology, Suzhou 215009, China; gengyh@szjl.com.cn (Y.-H.G.); wangruiyq@szjl.com.cn (R.W.); 5 Changchun New Industries Optoelectronics Technology Co., Ltd., Changchun 130103, China; xiaohd@cnilaser.com; 6 School for Optoelectronic Engineering, Zaozhuang University, Zaozhuang 277160, China

**Keywords:** gas sensing materials, vacuum technology, measurement system, optical and electrical responses

## Abstract

Gas sensing performance characterization systems are essential for the research and development of gas sensing materials and devices. Although existing systems are almost completely automatically operated, the accuracies of gas concentration control and of pressure control and the ability to simultaneously detect different sensor signals still require improvement. In this study, a high-precision gas sensing material characterization system is developed based on vacuum technology, with the objective of enabling the precise and simultaneous measurement of electrical responses. Because of the implementation of vacuum technology, the gas concentration control accuracy is improved more than 1600 times, whereas the pressure of the test ambient condition can be precisely adjusted between vacuum and 1.2 bar. The vacuum-assisted gas-exchanging mechanism also enables the sensor response time to be determined more accurately. The system is capable of performing sensitivity, selectivity, and stability tests and can control the ambient relative humidity in a precise manner. More importantly, the levels of performance of three different optical signal measurement set-ups were investigated and compared in terms of detection range, linearity, noise, and response time, based on which of their scopes of application were proposed. Finally, single-period and cyclical tests were performed to examine the ability of the system to detect optical and electrical responses simultaneously, both at a single wavelength and in a spectral region.

## 1. Introduction

Gas sensors have a broad application prospect in the fields of safety and environmental protection [1], health care [2], smart homes [3], etc. However, despite these opportunities, gaps persist in our knowledge regarding gas sensing technology. More specifically, whereas research studies on gas sensors have focused on the materials [4,5] and sensor structures [6,7], there has been less attention on the study of techniques for characterizing gas sensing properties. Furthermore, different groups of researchers tend to build test systems based on their own experiences, making it difficult to compare the findings between different studies because of discrepancies in the many technical details involved in the testing processes.

Although decades of development have resulted in the automation of gas sensor test systems, there remains much potential for improvement in terms of gas environment control and sensor-signal testing technologies. In general, there are two widely used modes of gas deployment in a measurement process, i.e., dynamic mode and static mode. For the dynamic mode [8], the target gas concentration is controlled by the setting of the ratio of the flow rate of the target gas to that of the balancing gas. This mode has three main drawbacks. (1) The flow control accuracy of a mass flow controller reaches ±1% of the full scale, such that it is difficult to realize accurate gas concentration control. (2) The test chamber should be small and simple enough. Otherwise, the duration for gas replacement is long and hence is averse to the determination of response and recovery times of the sensor sample. (3) It is difficult to accurately control the total pressure inside the test chamber, and hence, it is inconvenient to simulate the gas sensor performance associated with different altitude environments. For the static mode [9], the target gas was injected into an enclosed sensor test chamber pre-filled with balancing gas. The target gas concentration control accuracy of the static mode is even worse. The test chamber should be large enough for significant dilution of the target gas in a low concentration test. Furthermore, it is difficult to control ambient pressure and humidity during the test. In a previous study, we developed a quasi-static mode involving the use of a vacuum technique. In the system, different gases were injected into a pre-evacuated mixing chamber sequentially to prepare a gas mixture sample. Such a system has better gas concentration control accuracy and is compatible with very complex test chambers, and also can perform tests in different ambient pressures. However, the early quasi-static approach was quite primary, whereas we kept introducing new functions to improve the gas concentration control accuracy. The control strategy on water vapor (relative humidity) and volatile organic compounds (VOCs) are also improved. Considering that many sensors are susceptible to humidity change and have poor selectivity [10,11], study on the enhancement of the ambient humidity control and introduction of gaseous interferents in the testing system is of great importance.

Referring to the sensor-signal testing techniques, existing systems usually focus on the singular measurement of individual physical parameters, such as electrical properties [8,12,13,14], optical properties [15,16,17,18,19], thermal properties [20], surface acoustic waves (SAW) [21], and photoacoustic spectra (PAS) [22]. On the other hand, fewer systems are capable of the simultaneous measurement of multiple signals [23]. Several gas sensing materials, such as WO_3_ [24,25,26], V_2_O_5_ [27,28,29,30], NiO [31,32], and Pd [33,34], not only produce electrical responses but also undergo changes in transmittance, reflectivity, and dielectric constant, among other parameters, when encountering a specific gas. Hence, the simultaneous testing of multiple parameters, particularly electrical and optical signals, is of great importance to the research of the response mechanisms of these materials and to improve the gas sensing performances. For example, the electrical signal of WO_3_ is very sensitive to low concentration hydrogen but is prone to saturate below 1% [35]. However, its optical gas sensing response is linear to a hydrogen concentration above 10% [36,37,38], but fails to respond below 0.1%. The optical and electrical signals are complementary, allowing one to develop a “hybrid” hydrogen sensor in the sense of being capable of responding to a wide range of gas concentrations by making use of just one sensing element. The device is small and suitable to be used in hydrogen fuel cell powered vehicles. However, such a sensor is also expensive and not as robust as catalytic combustion sensors and metal oxide sensors, due to the implementation of optical parts.

In this work, a gas sensing material characterization system focusing on precise and simultaneous measurement of optical and electrical responses was developed based on our previous work [39]. The system design and selected components are disclosed in every detail. In its current form, the gas-concentration control accuracy is further improved by more than 1600 times compared to that of the one based on dynamic mode. The three-level flow control method makes the gas mixing procedure become more efficient and accurate. The control strategies on gaseous interferents such as water vapor and VOCs are also discussed with the aid of two case studies. To improve the test accuracy of electrical signals, all components, including meters, cables, and connectors, were carefully selected, and the leakage current was conducted directly to the circuit common, which reduces the lower limit of the current signal to the picoamp (pA) level. For the optical signal test, we designed three optical power test structures; compared their detection power ranges, linearities, response times, and noise levels; and determined their advantages, disadvantages, and applicable scenarios. Finally, the ability of the system to simultaneously detect optical and electrical signals at a single wavelength and in a specific spectral range was verified through different experiments. Compared with existing gas sensing material characterization systems, our proposed system adopts a modular design and is easy to expand; the user can measure radio frequency (RF) signals, reflectance spectra, and other signals by changing the relevant connectors or flanges. This system can also be used in conjunction with gas chromatography and mass spectrometry, for studies on gas sensing mechanisms.

## 2. System Introduction

### 2.1. System Modules and Parts

**Figure 1:**
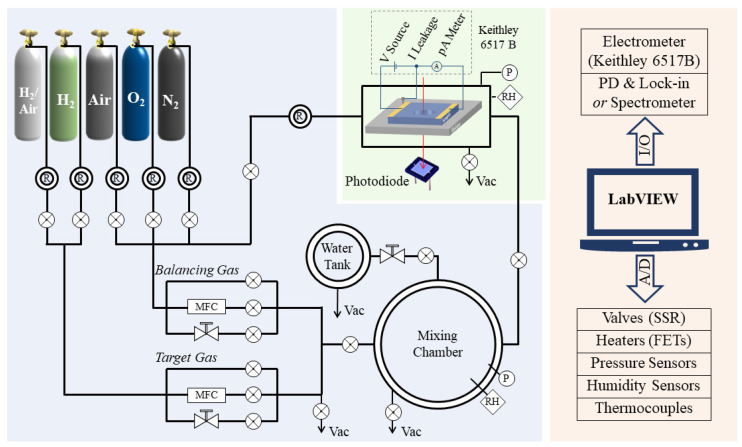
Schematic diagram of gas sensing material characterization system.

Figure 1 shows a schematic diagram of the system, which consists of three modules, i.e., gas-mixing module, sensor test module, and system control module.

The gas-mixing module is used for the target-gas preparation. This module is composed of gas cylinders, regulators, pipelines, mass flow controllers (MFCs), high-precision needle valves, a humidity-control chamber, and a gas-mixing chamber. The pipeline is an electrochemically polished 1/4” stainless-steel tube and is equipped with a spiral heating belt. By heating and evacuating the pipeline, the system can easily switch between different target and balancing gases. The flow-rate control for both the target and balancing gases is realized via a combination of an MFC, needle valve, and a vacuum-compatible electromagnetic (EM) valve. The MFC (EL-FLOW Base, Bronkhorst High-Tech B.V., Vorden, The Netherland) is used to adjust the flow rate between 0 and 200 standard cubic centimeters per minute (SCCM), whereas the needle valve (VMYS U-04T, Unilok Corporation, Icheon, Korea) provides flow regulation between several SCCMs and standard liter-per-minute (SLM) levels. The EM valves (SMC VX214LA and XSA series, SMC Corporation, Tokyo, Japan) are used for rapid filling directly into the chamber. The ON/OFF time for this valve is on the order of milliseconds and highly repeatable. The humidity-control chamber is a KF-40 three-way cross equipped with heating belt and thermocouple for the precise control of temperature, which ranges from room temperature to 100 °C. When the needle valve between the humidity-control chamber and gas-mixing chamber is adjusted to a certain setting, water vapor will slowly enter into the mixing chamber and humidify the mixing gas. The gas-mixing chamber is an ISO 200 vacuum chamber with an internal volume of 4256 cm^3^. It is equipped with two absolute pressure sensors (730 (0–1000 Torr) and 761 (0–1 Torr), Setra Systems, Inc., Boxborough, MA, USA) and a humidity sensor (HIH4000, Honeywell International Inc., Charlotte, NC, USA). These types of pressure sensors are unaffected by the type of gas and have an accuracy of ±0.25% reading, which is much more accurate than an MFC (±1% of full scale (F.S.)). 

The test chamber is a stainless-steel ISO 63 straight union, with four KF-40 flanges evenly distributed in the center. Its internal volume is 958 cm^3^. The chamber is equipped with a piezoresistive pressure sensor (5350 (0–1.6 bar), Setra Systems, Inc., Boxborough, MA, USA), a humidity sensor, and a thermocouple (CHAL-010, Omega Engineering, Inc., Norwalk, CT, USA). The chamber has a suspended heater with two bullet-type heaters inside (CSH-1011001, Omega Engineering, Inc., Norwalk, CT, USA) and a sheath-grounded thermocouple (SCASS-062G, Omega Engineering, Inc., Norwalk, CT, USA), for the accurate control of sample temperatures, which range from room temperature to 700 °C. The structure of the test chamber for optical signal tests is described in detail in Section 3. The DC signal of a sample is measured via a high-resistance electrometer (Keithley 6517B, Tektronix, Inc., Beaverton, OR, USA) with a built-in high precision voltage source (range: 0–1000 V, resolution: 5 mV) and a pA (10^−12^ A) meter (range: 20 pA to 20 mA, resolution: 0.1 pA). The 6517B electrometer is connected to a hermetically sealed triaxial connector (BJ77HS, Cinch Connectivity Solutions Co., Ltd., Waseca, MN, USA) mounted on a stainless-steel chamber via a low-noise triaxial cable (Keithley 7078-TRX-10, Tektronix, Inc., Beaverton, OR, USA). This cable has a graphite layer inserted between adjacent insulating layers, which effectively reduces the charges induced by cable movement or bending. The triaxial cable and the shielding of the stainless-steel chamber significantly reduce the noise induced by environmental electromagnetic interference. It is noteworthy that a silver-coated copper wire is used to connect the sample holder to the common of the electrical signal test circuit, i.e., the meter-low terminal of the 6517B. With this configuration, leakage current between the electrodes and the sample holder will be conducted directly to the circuit common and will not be counted as part of the sample current. When this set-up is used in conjunction with the constant voltage heating method (instead of PID temperature control) and a sheath-grounded thermocouple, we can measure the sensor signal down to the pA level.

Finally, we use LabVIEW programs for automatic measurement. With this program, the environmental parameters, such as temperature, humidity, and gas pressure, of the different chambers are measured by sensors and transmitted to a computer via an A/D interfacing card. The program compares the measured value to setpoints and then sends control signals to actuating components, such as the MFC, valves, pumps, and heaters, to ensure that the real values remain close to the setpoints. Meanwhile, the optical and electrical signals of the gas sensing materials are detected and transmitted back to the LabVIEW program and recorded simultaneously. Additionally, the test program can be easily configured to adapt to different test requirements, making the system ideal for high precision, multi-cycle automated testing.

### 2.2. Control of Gas Environment

**Figure 2:**
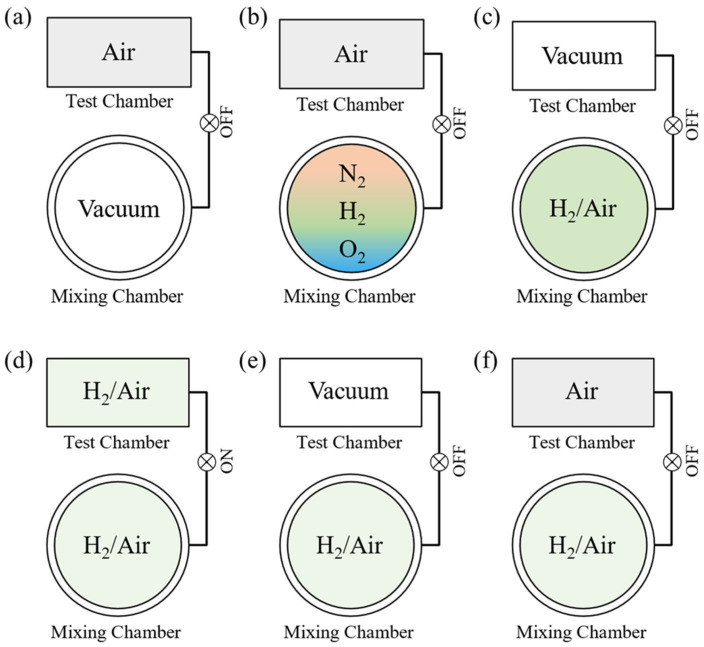
Schematic diagram of environmental gas control strategy. (**a**) shows the evacuation of the mixing chamber Schematically, while (**b**) depicts the gas mixing process, actually different gases are injected chronologically. (**c**) As soon as the target gas is prepared, the test chamber is evacuated. (**d**) Then the valve between mixing chamber and test chamber is opened to inject the target gas. (**e**) When the sample signal stabilizes in hydrogen, the test chamber is evacuated again, (**f**) then synthetic air is injected into the test chamber for recovery curve.

Accurate environmental gas control, including of its gas composition and total pressure, is a unique feature of our system. The gas environment control strategy is visualized in Figure 2. A typical test procedure consists of 6 steps, described as follows:

1. ***The mixing chamber is evacuated before the gas mixture is prepared***. At this stage, the test chamber is filled with synthetic air, which is prepared from 99.99% N_2_ and 99.99% O_2_. For the test, the system records the baseline of the sensor.
2. ***Different gas components are injected sequentially into the mixing chamber***. For this example, the desired preparation is a hydrogen–air mixture. To produce this gas mixture, high-purity hydrogen, nitrogen, and oxygen are introduced into the gas mixing chamber sequentially. Oxygen must be added last to minimize the risk of explosion. When the partial pressure of hydrogen reaches the setpoint, the pipeline is evacuated, and then the next gas (N_2_) is fed into the chamber. The partial pressure of each gas is monitored by two absolute-pressure sensors and is controlled by a combination of an MFC, needle valve, and EM valve. The concentration of each gas is then determined by its partial pressure. For example, if the partial pressures of hydrogen, nitrogen, and oxygen are 1 kPa, 78.21 kPa, and 20.79 kPa, respectively, then the hydrogen concentration is 1%. During the gas-mixing process, the sample is still soaked in synthetic air.
3. ***The air in the test chamber is evacuated.*** This stage of the process requires approximately 3 s.
4. ***The valve between the mixing chamber and test chamber is opened***. When the two chambers are connected, the gas expands freely from the mixing chamber to the test chamber. The pressures of both chambers stabilize in ~1 s. Because the volumes of the mixing chamber and the test chamber are 4256 cm^3^ and 958 cm^3^, respectively, the total pressure of the mixing chamber is adjusted to 1.225 bar during the mixing process. When the evacuated test chamber is connected to the mixing chamber, the pressure of the two combined chambers reaches exactly 1 bar.
5. ***The test chamber is evacuated.*** When the sample signal stabilizes in hydrogen, the test chamber is evacuated again, in preparation for synthetic air.
6. ***Synthetic air is injected into the test chamber.*** The test chamber is directly filled with synthetic air at a pressure of 1 bar for rapid gas exchange. Thereafter, the gas mixing chamber is evacuated again for the next cycle of gas preparation.

The gas environment control strategy of our system is different from the traditional dynamic mode and static mode; this new mode is referred to as a quasi-static mode. Unlike the dynamic mode, which is presently the most widely used, our system is developed based on vacuum technology, which results in four improvements to our system.

First, through the addition of a vacuum-compatible mixing chamber, the gas-mixing strategy is changed from continuous and simultaneous flow to intermittent and sequential flow. In dynamic mode, different gases flow continuously and simultaneously, and the only control variable for gas concentration is the relative mass flow rate, as shown in Equation (1). In this equation, *c*_1_, *c*_2_ and *f*_1_, *f*_2_ represent the relative concentrations and mass flow rates of gas_1_ and gas_2_, whereas *t* signifies the duration of the gas flow. By contrast, our system uses intermittent and sequential flows for different gases. Thus, we can further adjust the gas injection times (*t*_1_, *t*_2_ in Equation (2)) to control the gas concentration ratio. In other words, in our quasi-static mode, there are two control variables for gas concentration control, i.e., flow rates and injection times. More importantly, their concentration control capacities are multiplied. Therefore, the dilution capacity of this system is greatly enhanced.

(1) $$\frac{C_{1}}{C_{2}}=\frac{f_{1}\times t}{f_{2}\times t}=\frac{f_{1}}{f_{2}}$$

(2) $$\frac{C_{1}}{C_{2}}=\frac{f_{1}\times t_{1}}{f_{2}\times t_{2}}=\frac{f_{1}}{f_{2}}\times \frac{t_{1}}{t_{2}}$$

The second improvement is the change in the gas concentration judgement standard from the mass flow rate in dynamic mode, to the partial pressure in our quasi-static mode. The two absolute-pressure sensors used in our system are independent of the gas type and have an accuracy as high as ±0.25% of the reading. For comparison, the mass flow rate measured by MFCs is not only sensitive to the gas type, but also has a low accuracy (±1% F.S.). Therefore, the gas concentration judgement standard of the quasi-static mode is much more accurate than that of the dynamic flow mode.

**Figure 3:**
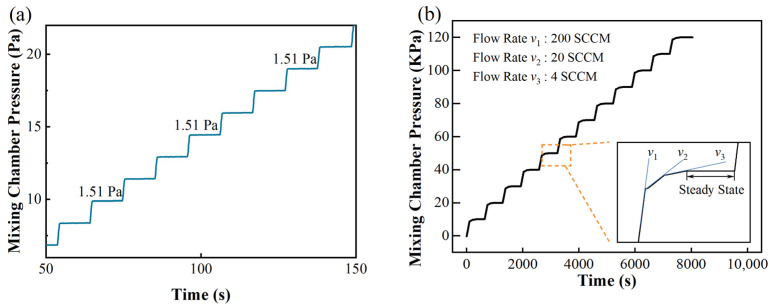
(**a**) Examination of partial pressure control limit and (**b**) three-level flow control method for precise and efficient partial pressure control.

The aforementioned two improvements lead to a significant increase in the accuracy of the gas distribution. The air dilution capacity of the dynamic mode and quasi-static mode are compared as follows. For example, given a system that is equipped with two MFCs (200 SCCM) for both the target and background gases, although the accuracy of the MFC is ±1% F.S., its minimum flowrate is 2% of the full scale, which is 4 SCCM for hydrogen. Therefore, in the dynamic mode, the maximum dilution rate is the maximum ratio of flow rates, i.e., 200/4 = 50. By contrast, for our system, when the mixing chamber has been evacuated, hydrogen flows into the mixing chamber at a minimum flow rate of 4 SCCM. The dilution capacity is determined by the minimum controllable partial pressure. We tested the pressure control limit by introducing hydrogen gas into the mixing chamber for 0.5 s every 30 s. As shown in Figure 3a, the chamber pressure increased stepwise. The increment ΔP in each step is about 1.51 ± 0.01 Pa. After the chamber is filled with N_2_ and O_2_, the total pressure reaches 1.225 bar, which indicates that H_2_ is diluted (122,500/1.51) = 81,126 times, which is more than 1600 times larger than in the dynamic mode. Taking into account both efficiency and accuracy, we used a three-level flow control method for large partial pressure control. The detail of this method is shown in Figure 3b. Mostly in a step the flow rate is set to be 200 SCCM. When the pressure difference between the present and target value is smaller than 1400 Pa, the flow rate decreases to 20 SCCM. When the pressure difference is smaller than 300 Pa, the flow rate further decreases to 4 SCCM. With this method, the real partial pressure can be controlled within ±0.1% of the setpoint in the pressure range of 10–120 kPa.

**Figure 4:**
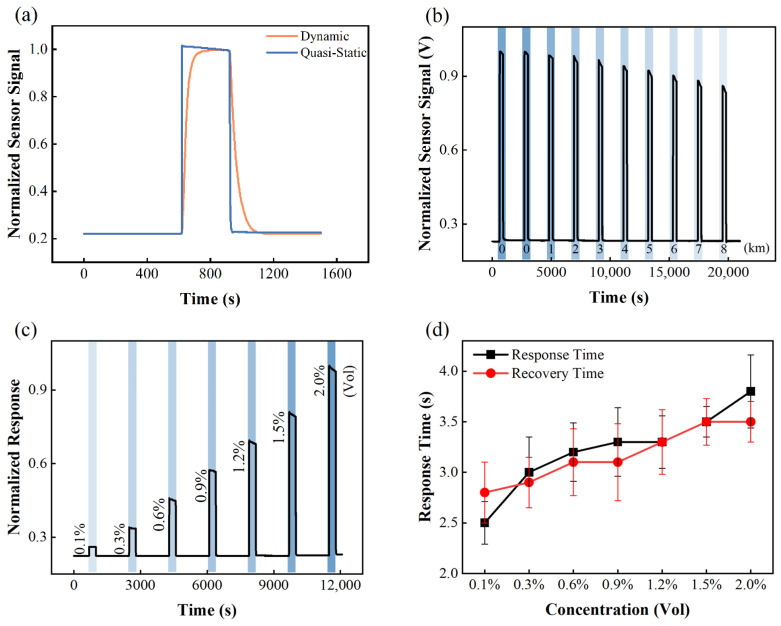
(**a**) Hydrogen sensor responses for conventional dynamic mode and vacuum-assisted quasi-static mode; (**b**) hydrogen sensor responses recorded at different ambient pressures, where hydrogen concentration in air is maintained at 2%; (**c**) hydrogen sensor responses recorded at different hydrogen concentration; (**d**) the response and recovery times plotted as functions of hydrogen partial pressure. The sensor used in this measurement is a commercialized catalytic combustion sensor. The working temperature is 250 °C and the applied voltage is 12 V.

The third improvement realized by the implementation of vacuum technology is the vacuum-assisted gas exchange scheme used in the test chamber, which greatly facilitates the determination of the response/recovery times of the sensors. With the proposed configuration, the hydrogen gas concentration in the test chamber can change from 0% to the target concentration within 1 s. By contrast, for the conventional dynamic mode, gas environment exchange relies on a very slow replacement process, and hydrogen concentration increases and decreases in a gradual manner. Figure 4a shows the response and recovery curves of a commercial hydrogen sensor (HNC-H2-2, NanoGrid Technology, Suzhou, China) in both the vacuum-assisted mode and conventional dynamic mode (flow rate: 1000 SCCM). The result shows that the response and recovery times tested using the vacuum-assisted mode are more accurate. It has been verified that the vacuum-assisted gas exchange process is suitable for all solid-state sensors such as metal oxide gas sensors and catalytic combustion gas sensors. However, it is noteworthy that the sensor signal showed a different trend in two modes. In vacuum-assisted mode, the sensor’s signal keeps decreasing after reaching a maximum value. In contract, the sensor signal increases abruptly and then gradually approaches the saturated value in dynamic mode. This is because in vacuum-assisted mode, the sensor is enclosed in the test chamber. On the other hand, the catalytic combustion sensor used in this test consumes hydrogen while working, such that the hydrogen concentration inside the chamber keeps decreasing as a result. By using the vacuum-assisted quasi-static mode we further measured the sensor’s responses against a different hydrogen concentration. Data are shown in Figure 4c. The response and recovery times are plotted against the hydrogen concentration in Figure 4d. For the catalytic combustion type hydrogen sensor, both the response time and recovery time increase with increasing hydrogen concentration. This performance is different from that of a metal oxide [40] and palladium metal-based hydrogen sensors [41]. Furthermore, if simultaneous measurement of electrical and optical signals is not required, we can use a much smaller test chamber, which will further accelerate the gas exchange process and minimize the gas pressure fluctuation. However, the vacuum-assisted method is not applicable to electrochemical sensors, thermal conductive sensors, and most optical-type sensors. For these types of sensors, our system also provides a closed circulation method and free diffusion method, which will be explained in future work.

Finally, the fourth improvement due to the use of vacuum technology is the ability to control the total gas pressure in the test chamber. Because the chambers and components are vacuum-compatible, the total pressure in the test chamber can be continuously adjusted between 1 Pa and 1.2 × 10^5^ Pa. Therefore, with this system, the ambient pressures at different altitudes, and even in a vacuum reactor, can be simulated. Figure 4b shows the responses of the hydrogen sensor to 2% hydrogen in air at different altitudes (0–8 km), wherein the total pressure varies between 0.3 and 1.1 atm, but the hydrogen concentration is always maintained at 2%. As shown in the graph, the sensor exhibits completely different response behaviors at different pressures: the lower the ambient pressure, the lower the sensor response. Therefore, it is of great practical importance to characterize the response behavior of the sensor at different altitudes.

### 2.3. Control of Gaseous Interferent

**Figure 5:**
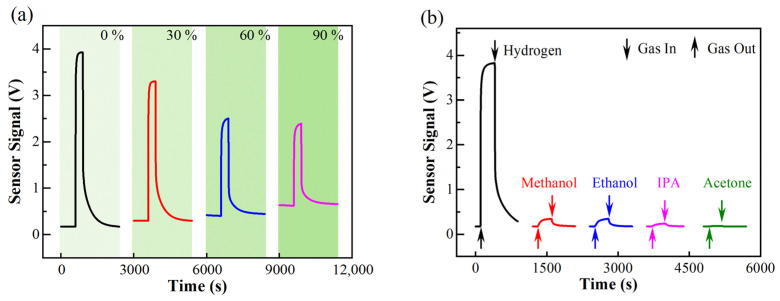
(**a**) Humidity dependance of the sensing response of Winsen MQ-8 sensor for 1000 ppm hydrogen. (**b**) Selectivity test of Winsen MQ-8 sensor measured against 200 ppm methanol, ethanol, isopropanol (IPA), and acetone. Both the heating voltage and the applied voltage for signal measurement are 5 V, corresponding to a working temperature ~250 °C.

In addition to precise target gas control, our system is also capable of controlling the concentration of many gaseous interferents, such as water vapor and volatile organic compounds (VOCs). Different from many existing systems using a water evaporator or bubbler to humidify the gas mixtures, our system takes water vapor as one of the components in the gas composition. Its partial pressure is controlled to set the relative humidity in the environment. In a typical process, the mixing chamber is first evacuated to base vacuum. Then, 100 Pa nitrogen is introduced into the chamber to reduce the physisorption of water molecules on the chamber wall. Next, water vapor is introduced into the chamber from the water tank shown in Figure 1. The partial pressure of the water is monitored using a capacitive pressure sensor. After the water vapor pressure reaches the target value, hydrogen, nitrogen, and oxygen are introduced into the chamber sequentially to generate a humid hydrogen-air admixture. The relative humidity is calculated using the absolute water vapor pressure and is monitored using relative humidity sensors mounted in the mixing chamber and test chamber. To illustrate the humidity control capability of our system, a commercialized metal oxide gas sensor (MQ-8, Winsen Electronics Technology Co., Ltd., Zhengzhou, China) was tested in 1000 ppm hydrogen under different levels of relative humidity. As shown in Figure 5a, the sensor baseline increases while the sensor signal decreases with increasing humidity level, which is consistent with the sensor specification. For metal oxide sensors, the resistance of the sensing element will decrease in humid conditions [42], since the sensor signal is the voltage measured across the load resistor, which is stable in humid conditions. Therefore, the sensor baseline increases as a result. Furthermore, less active sites responsible for adsorption and reaction hydrogen are left, such that a decrease in the sensor signal results [42].

Other gaseous interferents, such as methanol, ethanol, and other VOC vapors, are generated using a VOC generator (OVG, Owlstone Inc., Cambridge, UK). This apparatus has a permeation tube and a diffusion tubing system The contents of these interferents are also evaluated via their partial pressures in the sample gas. The VOCs generator should use synthetic air as carrier gas to realize accurate control on gas concentration. Figure 5b shows the response of a Winsen MQ-8 sensor to 1000 ppm hydrogen and 200 ppm methanol, ethanol, isopropanol, and acetone. Results show that although the sensor response to hydrogen is much larger than that of VOCs, the sensor response to VOCs is still nonnegligible. On the contrary, the selectivity of the catalytic combustion sensor shown in Figure 4 is much better. Meanwhile, the measurements successfully illustrate that our tungsten oxide-based hydrogen sensor exhibits much better selectivity compared to the Winsen MQ-8 sensor. It is recognized that the use of (1) chemo-selectivity or (2) appropriated working temperature are two effective ways for enhancing the selectivity in a gas sensing process. The former works better, especially when palladium is used as a catalyst or sensing materials.

## 3. Optical Signal Measurement

### 3.1. Optical Test Unit

**Figure 6:**
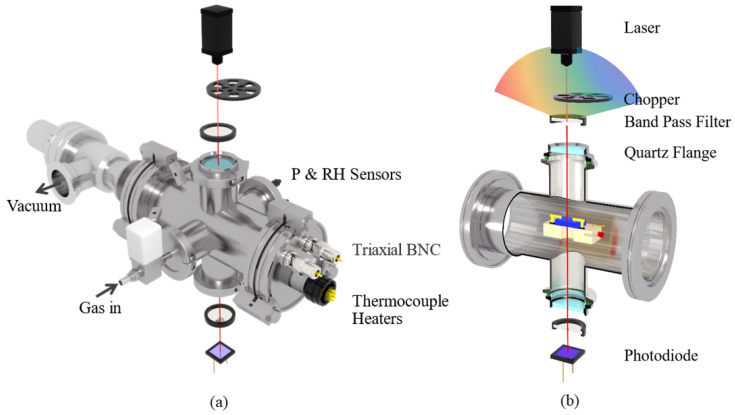
(**a**) Schematic diagram of test chamber and components used for optical test; (**b**) cross-sectional view of optical path and sample holder.

The simultaneous measurement of optical and electrical signals is another unique feature of this system. We can measure either single-wavelength transmittance or transmittance spectra in a certain wavelength region while obtaining high-accuracy electrical signal measurements. Figure 6a depicts a schematic diagram of the test chamber and the components used for the optical test. The main body of the test chamber is a stainless-steel ISO 63 union with four evenly distributed KF-40 flanges welded in the center. The outer surface of the front flange is equipped with 2 hermetically sealed triaxial BNC connectors and 1 hermetically sealed electrical connector. The former is used for the electrical signal measurement of the sample, whereas the latter is for sample heating and temperature control. The inner surface of the front flange is equipped with two stainless-steel screws penetrating through the whole test chamber. A sample heating block is suspended in the middle of the test chamber on the two screws, as shown in Figure 6b. The heating block is a polished 316 stainless-steel block with 2 heaters and a sheathed thermocouple inserted inside. There is a 5-mm-diameter through hole in the center of the heating block for optical signal measurements. Ceramic tubes are embedded between the test probes and the heater block, and between the stainless-steel screws and heater block, for electric and thermal insulation. The rear flange of the test chamber has a pneumatically controlled angle valve for chamber evacuation. The left flange, on which a high-vacuum EM valve (SMC XSA series) is mounted, is used for the air injection. To monitor the environmental parameters of the chamber, pressure sensors, temperature sensors, and humidity sensors are mounted on the right flange. The top and bottom flanges are equipped with highly transparent quartz flanges for optical signal measurements. The quartz flange can be replaced with an MgF_2_ flange for infrared and ultraviolet signal tests.

The optical test unit for the single-wavelength transmittance test is shown in Figure 6b. As shown in the diagram, a 785-nm laser is placed at the top, and the emitted laser is modulated via a chopper to 190 Hz. The laser then passes through a 785-nm bandpass filter and directly shines onto the sample via the top quartz flange. After passing through the bottom quartz flange and bandpass filter, the laser is then detected by a photodiode (PD) sensor. The signal of the PD is detected by a lock-in amplifier with a reference frequency the same as that of the chopper. It should be noted that: (1) In the diagram, the two 785-nm bandpass filters are suspended in the air to help the readers of this paper to conveniently understand the light path. In practice, the upper filter is mounted directly on the top quartz flange, whereas the lower filter is fixed onto the PD. With these special arrangements, the influence of ambient light is greatly eliminated even without the aid of a chopper and lock-in amplifier. (2) This diagram shows only a typical test structure. The components used for optical tests can be replaced according to the test requirements. For example, the laser can be replaced with a tungsten halogen lamp for spectroscopic measurements. The bandpass filter can be replaced with a polarizing filter for polarized light measurements. The PD can also be replaced with a pyroelectric detector for high-frequency laser tests. Moreover, the sample holder can be tilted 45° to enable reflectance measurements at the left flange.

### 3.2. Calibration of Optical Components

In this study, we focused on three light-intensity test methods. Before a detailed comparison, which is presented in Section 3.3, we have to calibrate the optical components. The following calibration process should be performed periodically once a month for system maintenance.

**Figure 7:**
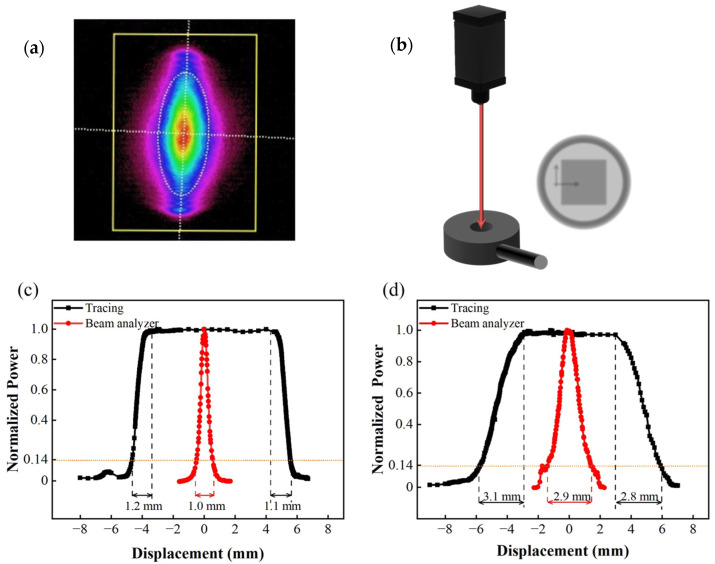
Calibration of spatial distribution of laser power: (**a**) laser spot intensity map; (**b**) sketch of laser spot dimension measurement setup; (**c**) horizontal power distribution; (**d**) vertical power distribution.

First, the size and position of the laser spot should be carefully controlled during tests, because the hole in the center of the sample holder for optical signal measurement is only 5 mm in diameter. Because of the possibility of vibration in the sample holder during evacuation, the spot size should preferably be less than 2 mm to avoid being blocked by the sample holder. The spot size of the laser has been factory calibrated using a laser beam profiler (Spiricon, SP620U, Ophir Optronics Solutions Ltd., Jerusalem, Israel), and the result is shown in Figure 7a. The horizontal and vertical distribution of the intensity is shown in the red curves of Figure 7c,d. However, although this method is the most accurate, the beam profiler is expensive and unsuitable for onsite calibration. For this reason, we designed a manual test setup for laser spot size evaluation, as shown in Figure 7b. The set-up is composed of a thermopile (TP) detector (3A-SH, Ophir Optronics Solutions Ltd., Jerusalem, Israel) and an XY axis manual displacement platform. The TP detector has a 10 × 10 mm^2^ effective detection area. Before measurement, we first move the laser spot out of the sensitive region of the TP detector. We then slowly move the laser spot onto the thermopile detector using the XY platform. Once the spot partially shines on the TP detector (as shown in Figure 7b), the reading of the power meter starts to increase from zero. Once the laser spot is completely inside the detector, the optical power meter reading reaches a maximum value. The reading will be maintained at its maximum for as long as the spot remains in the sensitive region. As the spot continues to move in the same direction and gradually moves outside the detector, the light intensity starts to decrease again and eventually reaches zero. The displacement over which the light intensity starts to increase (or decrease) until it reaches its maximum (or minimum) value is the size of the laser spot. Based on observations from moving the spot in both the vertical and horizontal directions, the width and length of the spot can be determined. As illustrated by the black curves in Figure 7c,d, the average lateral and longitudinal spot sizes measured using this method are 1.15 and 2.95 mm, respectively, which are consistent with the results obtained using the beam profile (1.0 and 2.9 mm, respectively). Based on this result, we decided to mount a 2-mm aperture on the exit of the 785-nm laser to limit the spot size.

**Figure 8:**
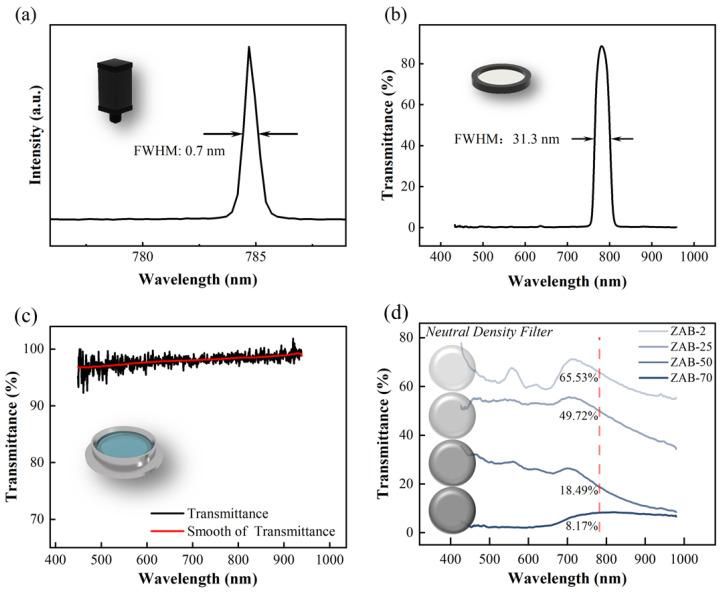
Spectrum test of optical components: (**a**) laser spectrum; (**b**) transmittance spectrum of bandpass filter; (**c**) transmittance spectrum of quartz flange; (**d**) transmittance spectrum of neutral density filter.

Spectrum tests were performed on the optical components; the results are shown in Figure 8a–d. The laser spectrum was measured using an Ocean Optics HR2000 spectrometer, and the results show that the semiconductor laser has a central wavelength of 748.8 nm and a full width at half maxima (FWHM) of ~0.7 nm, which indicates that the laser is of good monochromaticity. The transmittance spectra of the bandpass filter, quartz flange, and neutral density filter were obtained using the Ocean Optics QE65000 spectrometer with an HL-2000 white light source; the results are shown in Figure 8b–d. The transmittance spectrum of the bandpass filter (Figure 8b) shows a central wavelength of 780 nm and an FWHM of ~31.3 nm, which guarantees good shielding of ambient light. Figure 8c shows that the transmittance of the quartz flange is better than 96% in the wavelength region 400–900 nm, which indicates that the flange is suitable for optical measurements in the visible and near-infrared ranges. We also measured the transmittance spectrum of four neutral density filters. Figure 8d shows that the transmittance of the filters at 785 nm are 65.53%, 49.72%, 18.49%, and 8.17%, respectively. These filters were used in the following study on light-intensity test units.

### 3.3. Study on Light-Intensity Test Units

The measurement of the spectral responses of gas sensing materials is relatively simple compared to tests of light intensity at single wavelengths, especially when a fast response and a low-intensity measurement are required. In this study, we focused on the testing of three different light-intensity test units.

It is essential to select appropriate light-intensity detectors for the intended purpose. The three commonly used light-intensity detectors are the thermopile (TP), photodiode (PD), and pyroelectric detectors. A TP detector is composed of a series of circular, distributed bimetallic junctions. A laser striking on the sensor surface causes a temperature gradient, which results in a thermoelectromotive force related to the light intensity. This sensor is extremely stable and insensitive to ambient temperature. The operation range is 200 nm to 20 μm with laser power ranging from microwatt to hectowatt. On the other hand, the PD detector, most commonly the silicon-based PD, is a photosensitive p-n junction. Under zero bias or reverse bias voltage, a laser irradiated onto the photodiode will result in a photocurrent proportional to the light intensity. The current is then detected via measurement of the potential difference across a load resistor. Si-based PD can operate from 190 nm to 1100 nm within power ranges of picowatts to microwatts. Finally, a pyroelectric sensor uses a pyroelectric crystal that generates an electric charge proportional to the heat absorbed. This charge then charges a capacitor in parallel with the crystal, generating a voltage difference that is proportional to the pulse energy. This detector is particularly suitable for high-frequency optical signal measurements up to 10 kHz but is less durable and much more expensive than TP detectors.

For the characterization of the optical responses of gas sensing materials, a low-frequency, low-power laser signal is preferable. For this reason, in this study, we focused on only the TP and PD detectors. We further selected two fully validated readout devices, i.e., a high-precision data acquisition (DAQ) card and lock-in amplifier, to measure the signals of the PD detectors. Therefore, we were able to examine three different light-intensity test units: (1) TP detector and corresponding power meter, (2) combination of PD and lock-in amplifier, and (3) combination of PD and DAQ card. To compare the levels of performance of the three units, we first tested the linear range and linearity of the TP and PD detector. We then applied two different laser intensities, i.e., 10 mW and 0.8 mW, to investigate the response times and noise amplitudes of the three test units via an ON/OFF response test. A computerized 785-nm laser was used as a light source. All sensor signals were normalized for ease of comparison.

#### 3.3.1. Thermopile Sensor and Power Meter

**Figure 9:**
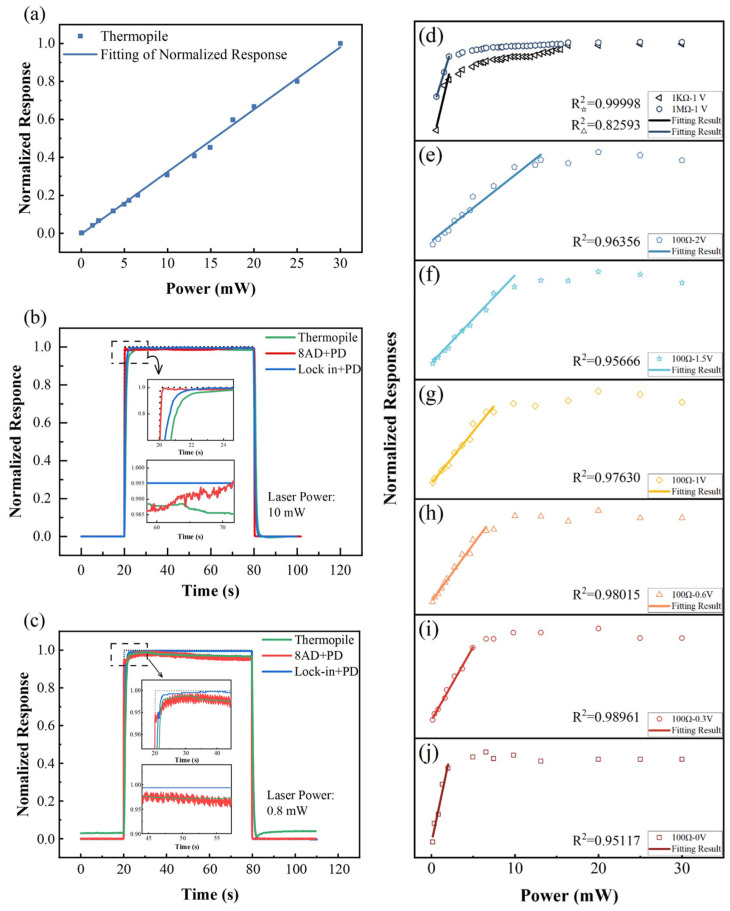
Comparison of levels of performance of three different optical power test units. (**a**) Linearity test of thermopile detector and power meter combination, (**b**,**c**) ON/OFF response test of three test units at 10 W and 0.8; (**d**–**j**) optical responses of photodiode detector for different bias voltages and load resistors.

For the TP detector, we used the Ophir 3A-SH high-accuracy detector and Ophir Nova power meter as an optical power test unit. Figure 9a shows the response of this combination to lasers with power values ranging within 0–30 mW. The blue line illustrates the linear fitting of the test results. The goodness of fit is R^2^ = 0.99791, which indicates that the TP sensor has excellent linearity for the entire test power range. In fact, the detection range of the 3A-SH TP detector is 10 µW to 3 W, and the linearity is less than ±1% according to the specification, which is far beyond our test requirements. In the ON/OFF response test, the response time (t_90_) of the TP detector with the meter was ~1.5 s for both 10 W and 0.8 W. This response time was the longest observed among three tested units, as shown in Figure 9b,c. For TP sensors, the absorbed laser power must be absorbed by the sensing element so as to build up a temperature gradient to result in a signal detected by a stack of thermopiles. The photothermal conversion process is relatively slow, whereas retardation of response is generally seen. This test unit had a medium noise level; the standard deviations of the normalized test signals at saturation were 0.0024 and 0.0052, respectively.

#### 3.3.2. Photodiode and DAQ Card

For this part of the study, the Hamamatsu S1337-1010BQ Si-based photodiode (PD) was selected. The potential difference across the load resistor was read using a YAV 8AD data acquisition card, which includes a 24-bit analog-to-digital converter. The saturation photocurrent of the PD detector at zero bias is typically approximately 1 mA, which corresponds to a laser intensity of 2 mW. Applying the appropriate reverse bias voltage will increase the response speed and enlarge the linear region of the PD sensor [33]. Meanwhile, the load resistance has a significant effect on the response and response time of the PD test unit [33]. Therefore, three load resistances (100 Ω, 10 KΩ, 1 MΩ) and six bias voltages (0 V, 0.3 V, 0.6 V, 1 V, 1.5 V, 2 V) were tested in orthogonal experiments on the optical response of the PD test unit. The results are shown in Figure 9d–j.

For the experiments on the 10 KΩ and 1 MΩ load resistors, the linear ranges of the response curves are very narrow at all bias voltages, as illustrated by the two typical curves shown in Figure 9d. The possible reason for this observation is that the potential across the high resistance load induced by the photocurrent was sufficiently large to balance out the reverse bias voltage applied by the external power supply. Therefore, the bias voltage failed to tune the linear region of the test unit when the high-resistance load was used. When the load resistance was decreased to 100 Ω, the modulation effect of the bias voltage became significant. As shown in Figure 9e–j, the linear region of the test unit expanded as the reverse bias voltage was increased. In particular, the linear region increased from 2 mW at zero bias voltage, to 12.5 mW at 1.5-V bias voltage. When the reverse bias voltage was increased further from 1.5 V to 2 V, the linear region remained the same. This is because the quantum efficiency of the photodiode, i.e., the number of electron-hole pairs generated per incident photon, increases with reverse bias voltage at first and then saturates at a certain value [43]. Finally, we performed experiments using 1.5 V reverse bias voltage to test the response time and noise level of the PD and DAQ card combination. As shown in Figure 9b,c, this test unit exhibited the shortest response times (0.13 s and 0.10 s) and largest noise levels at both 10 mW and 0.8 W. The standard deviations of the test signals were 0.0028 and 0.0102, respectively, in the steady state, approximately two times larger than those of the TP detector unit at 0.8 W. The fast response time is due to the simple structure of the readout circuit, in which the signal of PD is directly transmitted to the DAQ card and measured via a 24-bit analog-to-digital converter (ADC). On the contrary, the noise is relatively large because the noisy dark-current and background-current cannot be eliminated from the signal, and the ADC cannot perform sophisticated data processing [43].

#### 3.3.3. Photodiode and Lock-in Amplifier

Finally, the DAQ card was replaced with a combination of a lock-in amplifier and chopper. ON/OFF switching tests at 10 mW and 0.8 mW were performed; the results are shown in Figure 9b,c. The reverse bias voltage and load resistor were 1.5 V and 100 Ω, respectively. To achieve a balance between the noise level and response time, the sensitivity and time constants of the lock-in amplifier were set to 1 V/μA and 300 ms, respectively. As shown in Figure 9b,c, the response times at 10 W and 0.8 W were 1.05 s and 0.95 s, respectively, which were faster than those of the TP detector, but much slower than those of the DAQ card. For the lock-in amplifier, the data processing procedure is much more complicated than that of a DAQ card, which certainly has a longer response time. Furthermore, the noise level of the PD and lock-in combination was significantly lower than those of the other two test units. The standard deviations of the steady-state responses at different power levels were both 0.001, which was one-tenth that of DAQ and one-fifth that of the thermopile sensor at 0.8 W. The ultralow noise is because the noisy dark-current and background-current are eliminated from the signal of the PD by using a phase sensitive detector and further smoothed using a set of notch filters and low pass filters.

#### 3.3.4. Summary of Findings on Light-Intensity Units

Based on these results, the following conclusions can be drawn:

First, the TP detector exhibited the slowest response speed and a moderate noise level in the low-power test. Such a test unit is not suitable for gas sensing materials with optical responses faster than 2 s and are also not suitable for the accurate measurement of signals less than 1 mW. However, the TP detector has many advantages, such as an extra-large linear region, excellent stability, great linearity, and insensitivity to spot size and position. Combined with neutral density filters, the TP sensor is an ideal choice for the periodic calibration of optical components.

Second, the combination of a PD and DAQ card had the fastest response, but its noise level was relatively large and, hence, is not suitable for low-power tests, i.e., <1 mW. However, if the light intensity signal is approximately 10 mW, its noise level is only 14% higher than that of a TP detector. The price of the PD and DAQ combination is much lower than those of the other two units: approximately $200 for a single-channel unit and $100 per channel for eight channels. Furthermore, this combination is easy to operate, making it the perfect choice for beginners and multi-channel optical testing.

Finally, the combination of a PD and lock-in amplifier exhibited a relatively fast response time of ~1 s and an extremely low noise level. Therefore, this combination is the best choice for low-power tests. However, this combination is quite expensive, at approximately $8000 for a single channel. Additionally, the lock-in amplifier is sophisticated and less durable than a DAQ card. The operators must be well-trained before using this test unit.

## 4. Capability for Simultaneous Measurement of Electrical and Optical Signals

**Figure 10:**
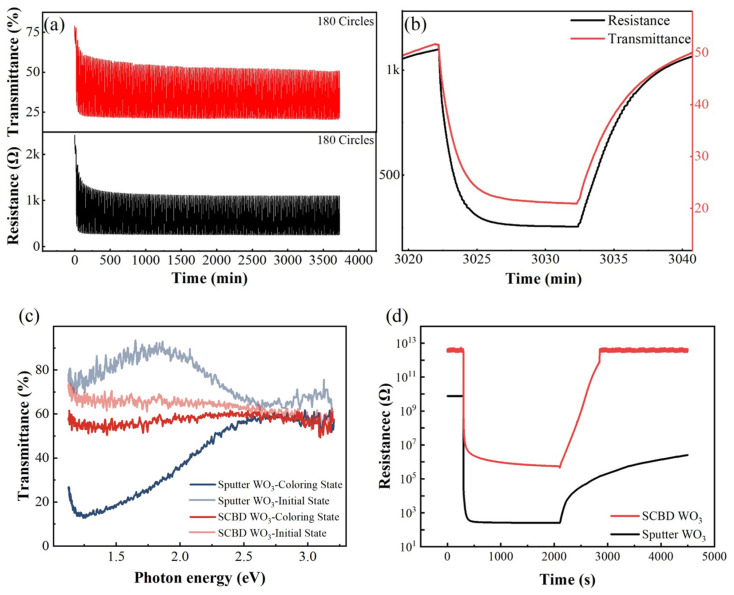
Simultaneous measurement of electrical and optical signals: (**a**) cyclical test for transmittance and resistance responses of sputtered WO_3_ film to 2% H_2_–air admixture at 80 °C; (**b**) enlarged view of 150th cycle of data shown in (**a**); (**c**,**d**) spectroscopic and resistive responses of sputtered WO_3_ film and SCBD WO_3_ film to 2% H_2_ in air measured at 80 °C. The applied voltage for electrical signal measurements is 1 V.

The capability of our system for the simultaneous measurement of electrical and optical signals was verified via the following two experiments. In the first experiment, the resistance and optical transmittance responses of sputtered WO_3_ films to one atmospheric pressure 2% H_2_–air admixture were measured simultaneously in a cyclical manner; the results are shown in Figure 10a. Before the measurement, a Pd catalytic layer was coated onto a WO_3_ film via magnetron sputtering. The light source was a 785-nm laser, and the measurement set-up is the combination of a PD detector and lock-in amplifier. During the test, the WO_3_ film was exposed to 2% H_2_–air admixture and synthetic air alternately every 10 min. Figure 10a shows the test results for 180 cycles, whereas Figure 10b shows a detailed view of the 150th cycle. In the first few cycles, there were significant decreases in the upper and lower boundaries for both the electrical and optical signals. Furthermore, these decreases were highly synchronized. Because the absorptance of WO_3_ in the red and infrared regions is related to the concentration of the hydrogen species injected inside it can be concluded that the baseline drift is due to the shortage of dehydrogenation time. As a result, hydrogen gradually accumulates during each cycle and eventually reaches a dynamic equilibrium at this test frequency. Such an inference cannot be drawn if only an electrical signal is present. This is because if the electrical contact between the Pd catalyst and the WO_3_ film becomes increasingly intimate, such as in the case of inter-diffusion, the upper and lower boundaries of the resistance signal will also decrease. However, such a change in electrical contact will not result in a change in transmittance, and thus this possibility has been negated by simultaneous measurement.

Finally, the spectroscopic and electric responses of two different WO_3_ films to one atmospheric pressure 2% hydrogen-air admixture were recorded and compared in Figure 10c, d. The two WO_3_ films were prepared via magnetron sputtering and supersonic cluster beam deposition (SCBD). The first sample was fabricated at 350 °C and a sputtering pressure of 10 mTorr. With these fabrication parameters, the sample became relatively dense, with a porosity of ~8.34% and a thickness of approximately 122 nm [44]. By contrast, the WO_3_ film prepared via the SCBD method was composed of loosely stacked WO_3_ nanoclusters. The film porosity was ~66%, and the thickness was 140 nm. The SCBD WO_3_ film can be easily removed via finger rubbing. Before the test, both samples were sputtered with the same amount of a Pd catalytic layer. As shown in Figure 10c,d, the resistance of the sputtered WO_3_ sample in air was ~10^10^ Ω, whereas the SCBD WO_3_ sample had a higher resistance of ~3 × 10^12^ Ω due to its porous structure. For both samples, a sudden decrease in resistance occurred after exposure to hydrogen. The sensor responses, defined by R_air_/R_H2_, of the two sensors were 3.17 × 10^7^ Ω and 6.51 × 10^6^ Ω, respectively. However, their resistances in hydrogen, i.e., 500 Ω and 1 × 10^6^ Ω, respectively, exhibited great disparity. This indicates that the conductivity of the two samples, i.e., the amount of hydrogen injected, varies considerably. This hypothesis was proved by the responses shown in the transmittance spectra, which are presented in Figure 10c. The transmittance of the magnetron-sputtered WO_3_ films decreased by more than 60%, whereas the variation in transmittance of the SCBD WO_3_ film was only 10%. Based on the thicknesses [45] and porosities of the two films, the mass thicknesses of the films (under the assumption that the films were as dense as single-crystal WO_3_) were calculated to be 111.8 nm and 47.6 nm, respectively. Therefore, the ratio of the transmittance spectra changes (6:1) was much larger than the ratio of the mass thicknesses (2.35:1).

A possible explanation is as follows. Given that the sputtered WO_3_ films are much denser than the SCBD WO_3_ films, although hydrogen injection can easily occur for both samples, the concentration of oxygen species in the interior of the two films are distinctly different. This is because the hydrogen species can diffuse into the film via a double injection mechanism with the aid of a Pd catalyst, whereas oxygen can diffuse into the film only in gaseous form. Evidently, the concentration of oxygen species in the SCBD WO_3_ film was much larger than that in the sputtered WO_3_ film. Therefore, the equilibrium point for the SCBD WO_3_ film shifted towards the dehydrogenation direction, which resulted in a much smaller transmittance change. This hypothesis is supported by the results of the recovery test for the electrical signal in air; the results are shown in Figure 10d. As shown in the figure, the SCBD film recovered to its initial state within 15 min. By contrast, the sputtered WO_3_ film required approximately 11 h to recover its initial resistance. Moreover, the absorption peaks of the two samples exhibited a large discrepancy during hydrogenation, which indicates that the electronic structures of the two films are distinctly different. With the aid of first-principles calculations, the hydrogen sensing mechanisms of WO_3_ films with different compositions and structures can be further studied.

## 5. Conclusions

A high-precision characterization system for the simultaneous measurement of the optical and electrical responses of gas sensing materials was designed and established based on vacuum technology. The incorporation of vacuum technology changed the gas-mixing process from the less accurate mass flow ratio control to high-precision partial-pressure control. With the addition of the injection time as the second control variable, the dilution ability of our system became more than 1600 times larger than that of the traditional dynamic mode. The vacuum-assisted gas exchange mechanism allows the system to accurately determine response and recovery time. Moreover, the total pressure of the test ambient condition can be tuned between 1 Pa and 1.2 × 10^5^ Pa. For the optical signal tests, three different optical power test units were systematically studied in terms of linear region, linearity, response time, and noise level. The results show that the thermopile detector is the ideal choice for system calibration, whereas the combination of a photodiode and DAQ card is suitable for high-speed or multi-channel measurement. Meanwhile, the combination of a photodiode and lock-in amplifier is the best choice for high-precision low-power tests. Finally, the superiority of the simultaneous measurement of electrical and optical signals in the proposed hydrogen-sensing mechanism study was proved in two sets of experiments. The system is assembled with standard vacuum components and is compatible with several in-situ test units, including test units for impedance spectrum, mass spectroscopy, UV–Vis spectroscopy, and Raman spectroscopy, making the system an ideal piece of equipment for the study of sensing mechanisms.

## Data Availability

Not applicable.
